# Efficacy and Safety of Lumen Apposing Self-Expandable Metal Stents for EUS Guided Cholecystostomy: A Meta-Analysis and Systematic Review

**DOI:** 10.1155/2018/7070961

**Published:** 2018-04-12

**Authors:** Nikhil R. Kalva, Vishwas Vanar, David Forcione, Matthew L. Bechtold, Srinivas Reddy Puli

**Affiliations:** ^1^Internal Medicine, University of Illinois College of Medicine at Peoria, Peoria, IL, USA; ^2^Interventional Endoscopy Services, Gastrointestinal Unit, Massachusetts General Hospital, Harvard Medical School, Boston, MA, USA; ^3^Division of Gastroenterology and Hepatology, University of Missouri-Columbia, Columbia, MO, USA

## Abstract

**Background:**

Patients with acute cholecystitis are treated with early cholecystectomy. A subset of patients are unfit for surgery due to comorbidities and late presentation. Prompt gall bladder drainage (GBD) with percutaneous or endoscopic approach remains a viable therapeutic option for nonoperative candidates. Endoscopic ultrasound (EUS) guided transluminal gall bladder drainage (EUS-GBD) continues to evolve as an alternative approach to percutaneous drainage. With continued refinement in stent technology, lumen apposing self-expandable metal stent (LAMS) offers several advantages. We performed a pooled analysis on the efficacy and safety of EUS-GBD with LAMS in nonoperative candidates with acute cholecystitis.

**Methods:**

Extensive English language literature search was performed in Medline, Embase, Cochrane Central, and Google Scholar using keywords “endoscopic ultrasound”, “stent”, “gallbladder”, “acute cholecystitis”, and “cholecystostomy” from Jan 2000 to Dec 2016. Fixed and random effects models were used to calculate the pooled proportions.

**Results:**

Data was extracted from 13 studies that met the inclusion criteria (*n* = 233). Pooled proportion of technical success was 93.86% (95% CI = 90.56 to 96.49) and clinical success was 92.48% (95% CI = 88.9 to 95.42). Overall complication rate was 18.31% (95% CI = 13.49 to 23.68) and stent related complication rate was 8.16% (95% CI = 4.03 to 14.96) in the pooled percentage of patients. Pooled proportion for perforation was 6.71% (95% CI 3.65 to 10.6) and recurrent cholangitis/cholecystitis was noted in 4.05% (95% CI = 1.64 to 7.48). Publication bias calculated using Harbord-Egger bias indicator gave a value of −0.61 (95%  CI = −1.39 to 0.16, *p* = 0.11). The Begg-Mazumdar indicator for bias gave Kendall's tau *b* value of −0.42 (*p* ≥ 0.05).

**Conclusions:**

EUS-GBD with LAMS is a safe and alternative treatment modality for patients needing gallbladder drainage, with acceptable intraprocedural and postprocedural complications. However, due to the limited data and lack of direct comparison with other methods, further controlled trials are necessary to estimate the overall efficacy and safety and the role of EUS-GBD with LAMS in management of nonoperative patients with acute cholecystitis.

## 1. Introduction

Cholecystectomy remains first-line treatment for acute cholecystitis due to stone disease or malignant cystic duct obstruction in patients who are considered good surgical candidates. Early laparoscopic cholecystectomy performed within two days of clinical presentation has favorable clinical outcome and remains highly cost effective [1]. In acutely ill patients unfit for surgery, percutaneous gallbladder drainage (PTGBD) with prompt decompression has shown evidence of reducing the risk of severe sepsis, gall bladder perforation, or even death [2]. Since its introduction in 1980s, the technique of PTGBD has undergone refinements with acceptable perioperative complication rates. Despite its highly successful insertion and improvement in short-term mortality, drain related issues continue to negatively impact outcomes due to accidental dislodgement, pain and discomfort, and quality of life [3]. PTGBD remains widely used because of its ease of placement either as destination therapy or as two-staged approach for patients who are future candidates for cholecystectomy.

Endoscopic ultrasound (EUS) now has a well-established role in transmural drainage of extraluminal fluid collections from complicated pancreatitis with excellent success in the management of pseudocysts and walled-off necrosis (WON). Despite several shortcomings of the earlier stents, the availability of the lumen apposing metal stents or LAMS (Axios; Xlumena, Inc., Mountain View, California, USA) has furthered our capacity to perform successful transmural drainage under EUS and image guidance [4]. The practical advantages of larger flanges at the end are the following: when fully expanded they permit excellent tissue apposition and resist stent migration. The larger inner diameter facilitates adequate drainage and a silicone covering assists in maintaining adequate seal between the gall bladder and the bowel. Additional advantages of the silicone covering include resistance to tissue ingrowth aiding in subsequent stent removal following maturation of the cholecystoenteric fistula. With continued expansion of therapeutic EUS, alternative transmural drainage of the gall bladder is entertained to overcome the recognized challenges in PTGBD [5]. Kwan et al. first described the novel transgastric/transduodenal (transmural) drainage of the gall bladder under EUS guidance with subsequent series reporting clinical success by various authors using plastic biliary stents across the tract [6, 7]. Stent migration, bile peritonitis, and pneumoperitoneum became an apparent following tract dilation at the time of stent deployment, limiting its wider adaptation and clinical use. Self-expandable metal stents (SEMS) with flanges at the end could overcome the flaws from tract dilation and prevent spontaneous stent migration [8]. Finally, with significant advancements in the stent technology and remarkable success of LAMS in the management of pancreatic fluid collections, various authors now have assessed feasibility of transmural drainage of the gall bladder in both retrospective and prospective cohorts.

The aim of our analysis is to assess the efficacy and safety of EUS-GBD using the newer LAMS in the management of inoperable patients with acute cholecystitis.

## 2. Material and Methods

### 2.1. Study Selection

The study was conducted based on the Preferred Reporting Items for Systematic Reviews and Meta-analysis guidelines (PRISMA guideline) [9]. A study protocol was developed by the study team prior to the initiation of the study. Studies evaluating the use of EUS for gall bladder drainage and stenting with lumen apposing stents that reported clinical success and complications were selected. Articles were searched using Medline, Embase, Cochrane Central, and Google Scholar and limited to English language. The last date of the search was December 2016. The search terms used include endoscopic ultrasound, stents, acute cholecystitis, gall bladder, and cholecystostomy. The citations were imported into EndNote and duplicates were removed. Articles were reviewed by title, abstracts, and full texts by two independent reviewers (N.R.K, S.R.P). Differences were resolved by mutual consensus. The agreement among reviewers for the collected data was quantified using Cohen's alpha [10]. Data from the studies were extracted into Excel spreadsheet for further analysis and performing statistical analysis.

### 2.2. Statistical Methods

This study was performed by calculating the pooled proportions (i.e., pooled proportion of patients with primary outcomes). First, the individual study proportion of outcome was transformed into a quantity using Freeman–Tukey variant of the arcsine square root transformed proportion. The pooled proportion is calculated as the back transform of the weighted mean of the transformed proportions using the inverse arcsine variance weights for the fixed effect model and the DerSimonian–Laird weights for the random-effect model [11]. Forrest plots were drawn to show the point estimates in each study in relation to the summary pooled estimate. The width of the point estimates in the Forrest plots indicated the assigned weight to the independent study. The heterogeneity among studies was tested using Cochran's *Q* test based on inverse variance weights [12]. If *p* > 0.10, it rejects the null hypothesis that the studies are heterogeneous. The effects of publication and selection bias on the summary estimates were tested using both the Harbord-Egger bias indicator and Begg-Mazumdar bias indication [13, 14]. Funnel plots were also constructed to evaluate publication bias using the standard error [14].

### 2.3. Assessment of Study Quality

There are no internationally agreed quality reporting scales for use in systematic reviews and meta-analysis using observational studies. We used the Strengthening the Reporting of Observational Studies in Epidemiology (STROBE) guidelines as a template for assessment of quality of each study [15]. We identified key elements that could potentially introduce bias and include selection bias and confounding bias.

## 3. Results

**Figure 1:**
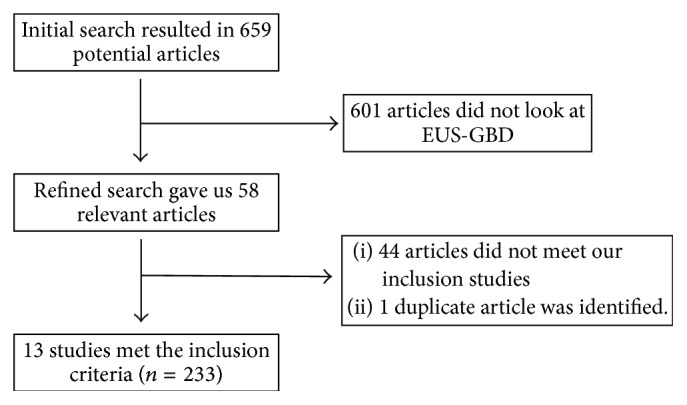
Flowchart of study selection.

The initial search identified 659 reference articles, of which 58 relevant articles were selected and reviewed. A total of 13 studies were selected for data extraction that met our inclusion criteria, with 233 patients included in the analysis [8, 16–27].  Figure 1 shows the flow diagram for the meta-analysis. The study characteristics are included in Table 1. The interrater reliability of the included studies using Cohen's Kappa gave a value of 0.81 suggesting excellent agreement among the study authors.

**Figure 2:**
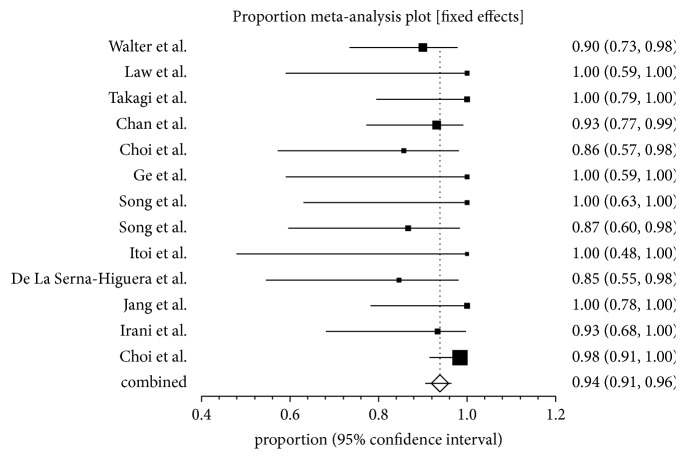
Forrest plot showing the individual study proportion of technical success in EUS-GBD.

**Figure 3:**
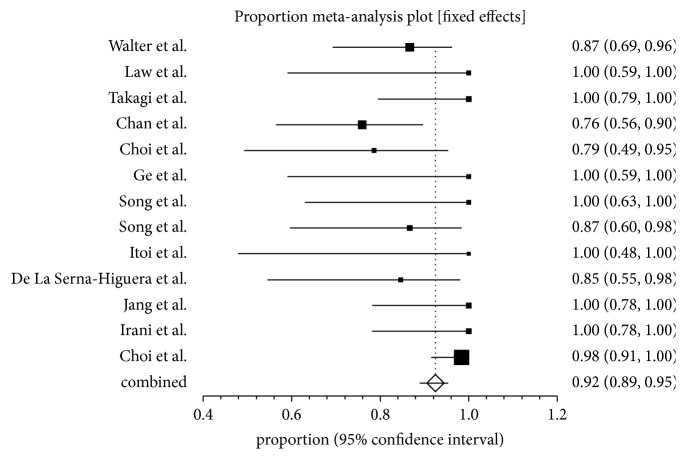
Forrest plot showing the individual study proportion of clinical success in EUS-GBD using fixed effect model.

The diagnosis of acute cholecystitis used in the studies was based on clinical and radiographic criteria. All the patients included in the reference studies were unfit for surgery. Patients with calculous and acalculous cholecystitis and malignant cystic duct obstruction were included in the analysis. Primary analysis of the study is predefined and include technical and clinical success and overall complication rates. The technical success is defined as successful stent deployment between the stomach or duodenum and the gallbladder. The pooled proportion of technical success using fixed effect model for endoscopic ultrasound guided gall bladder drainage (EUS-GBD) was 93.86% (95% CI; 90.56–96.49). Individual study proportions of technical success using fixed effect model are depicted in the Forrest plot and shown in Figure 2. The clinical success is defined as resolution of symptoms of acute cholecystitis following achieving successful gallbladder stenting. The pooled proportion of clinical success was 92.48% (95% CI; 88.90–95.42). Individual study proportions of clinical success using fixed effect model are depicted in the Forrest plot and shown in Figure 3.

**Figure 4:**
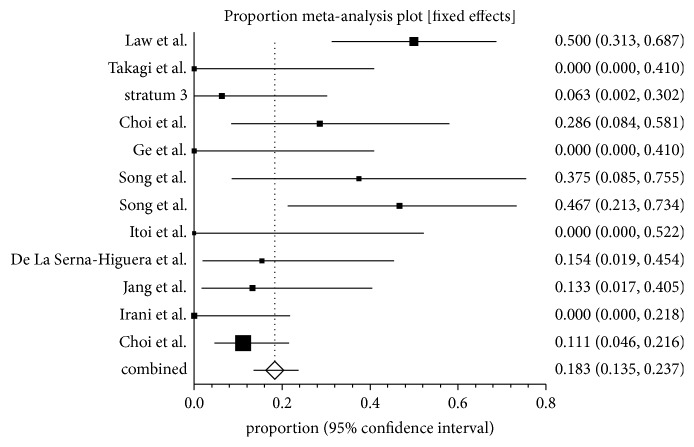
Forrest plot showing the individual study proportion of overall complications in EUS-GBD using fixed effect model.

The overall complication rate was 18.31% (95% CI; 13.49–23.68) and stent related complication rate was 8.16% (95% CI; 4.03 to 14.96) in the pooled percentage of patients. The individual study proportions of overall complications are depicted in the Forrest plot and shown in Figure 4. Pooled proportion for perforation was 6.71% (95% CI; 3.65–10.6) and recurrent cholangitis/cholecystitis was noted in 4.05% (95% CI; 1.64–7.48).

**Figure 5:**
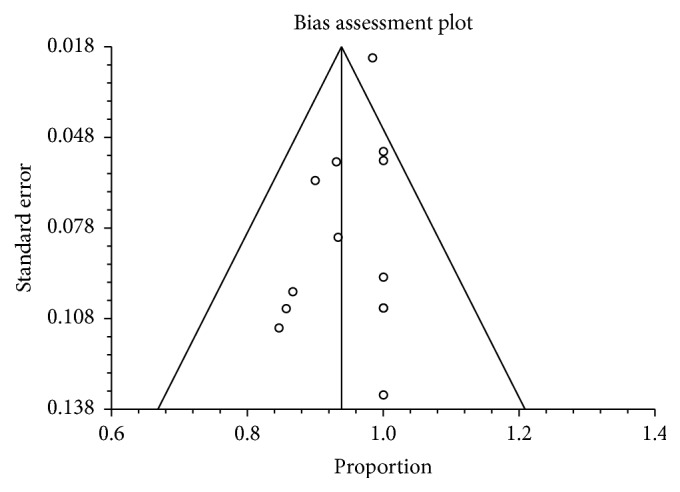
Funnel plot evaluating the effect of publication bias on studies investigating technical success in patients undergoing EUS-GBD.

Publication bias calculated using Harbord-Egger bias indicator gave a value of −0.61 (95% CI; 1.39–0.16, *p* = 0.11). The Begg-Mazumdar indicator gave Kendall's tau *b* value of −0.42 (*p* ≥ 0.05). Both these indicators showed that there was no publication bias. Additionally, Figure 5 represents funnel plot to assess and screen for publication bias for various outcomes that were analyzed for this study.

## 4. Discussion

EUS-GBD is a technically challenging procedure even with experienced endoscopists. Unlike walled-off pancreatic cysts including necrosis which are fixed to the gastric wall, the gallbladder is a mobile structure making the transmural puncture challenging. The technique consists of a multistep process which includes imaging of the gallbladder from the prepyloric antrum or the duodenal bulb [6, 7]. The body or the neck of the gallbladder is selected as an ideal site of entry over the fundus. The shortest distance from the enteral lumen to the gallbladder is selected and color flow Doppler is used to exclude interposing vessels. A 19-gauge needle is used to puncture after selection and, following needle entry, a guidewire (0.035 inch) is coiled into the gallbladder. Subsequently a cautery or mechanical track dilation is performed. An appropriate sized LAMS is selected and deployed from distal to proximal bowel wall under endoscopic and fluoroscopic control to achieve lumen apposition.

Our analysis is thus far the largest reported pooled data on the technical and clinical success rate in EUS-GBD with placement of LAMS with results approaching close to 95%. Most studies used Axios or BONA LAMS in the management. There are several advantages with use of LAMS in this setting when compared to use of either plastic or SEMS without the lumen apposing features. The large diameter of the LAMS stent facilitates adequate gallbladder drainage decreasing the risk of stent obstruction due to thick bilious or purulent secretions. Additionally, the prompt lumen apposition of the gallbladder with the lumen reduces the risk of bile leak peritonitis in addition to minimizing distal stent migration. With further refinement of the technique and stent technology, additional cholecystoscopy using smaller caliber or standard endoscopes with cholecystolithotomy could be considered [19]. The efficacy seems to be mostly in the immediate setting as long-term outcomes are not reported except in one study that was included in our analysis [18]. The study by Choi et al. assessed long-term efficacy outcomes of recurrent acute cholecystitis and need for reintervention after a median follow-up of approximately 275 days [18]. The rates of late stent related outcomes including distal migration and occlusion requiring reintervention were reported to be around 7.1% in the same study with relatively long-term stent patency rates close to 90% even at the end of 3 years in patients who survived the episode of acute cholecystitis. Subsequent management following formation of the mature cholecystoenteric fistula remains unclear for the published studies to draw a meaningful conclusion. The gallbladder can be accessed both from the prepyloric antrum and the duodenal bulb as described for successful stent deployment. Any drawn conclusions on the safety and efficacy remain unclear. In patients who have undergone PTGBD and remain unfit for future cholecystectomy, Law et al. have reported excellent rates of conversion to EUS-GBD with internalization with similar outcomes [23].

Transluminal drainage and stenting with LAMS have clear advantages in the management of these patients. Despite these important findings on efficacy, our analysis has also shown overall complications rates to be around 18% with direct stent and procedure related complication to be close to 8%. Though the overall complication rates are important they account for both the procedure related and independent outcomes. The more important outcome we believe is stent and procedure related outcome which appears to be acceptable. Recurrence rate of acute cholecystitis appears to be due to obstruction of the stent due to food material and is reported to be about 4% which could be managed with repeat endoscopy with manipulation to clear the stent of the debris. Other unusual stent related adverse events that are reported include self-limiting pneumoperitoneum, distal stent migration into the gall bladder, and tissue ingrowth at variable frequencies.

## 5. Limitations

Despite encouraging results in short-term clinical success with EUS-GBD and stenting in acute cholecystitis patients, the long-term efficacy of this approach in definitive management remains unclear as a subset of these patients remain indefinitely unfit for surgical cholecystectomy. The theoretical risk of entry of ingested food entry into the gall bladder with subsequent obstruction of the cystic duct and the common bile duct leading to acute cholecystitis and biliary sepsis remain with unknown frequency. This problem could be overcome by targeting the distal end of the LAMS to the neck of the gallbladder there by obstructing the cystic duct. This has an advantage by minimizing the entry of ingested food material into the common bile duct via the way of the cystic duct.

One of the major limitations of the analysis is the inclusion of only small retrospective case series from tertiary centers. The inclusion of small case series could greatly enhance the effect size compared to larger prospective studies. Additionally, the patients are carefully selected resulting in introduction of selection bias in the studies that were included. We systematically attempted to exclude the biases by performing Egger bias indicator and construction of the funnel plot. In our present analysis, we used both the Egger and Begg-Mazumdar bias indicators which showed no statistically significant bias [13, 14]

## 6. Conclusions

EUS-GBD using LAMS offers an alternative to PTGBD with good short-term technical and clinical success rate. Despite the reported complications, albeit managed conservatively, EUS-GBD using LAMS appears to be higher than most routine gastrointestinal endoscopic procedures. Further long-term prospective studies comparing its efficacy to transpapillary gallbladder stenting and mini-invasive strategies are needed before wide spread adaptation of this technique. In spite of the complexity of the procedure, it should be offered in highly specialized centers with the expertise in advanced therapeutic endoscopy with capacity to manage complications if they arise.

## Figures and Tables

**Table 1:** Study characteristics.

Author	Year	Number of patients	Study type	Type of stent
Walter et al.	2015	30	Prospective	Axios
Law et al.	2016	7	Retrospective	Axios
Takagi et al.	2016	16	Retrospective	BONA
Chan et al.	2016	25	Retrospective	Axios
Choi et al.	2016	14	Case control	Regular stent
Ge et al.	2016	7	Retrospective	Micro Tech
Song et al.	2010	8	Prospective	Regular stent
Song et al.	2012	15	Prospective	BONA
Itoi et al.	2012	5	Retrospective	Axios
Serna-Higuera et al.	2013	13	Prospective	Axios
Jang et al.	2012	15	Prospective	BONA
Irani et al.	2015	15	Retrospective	Axios
Choi et al.	2014	63	Retrospective	BONA
